# Chlorine Induces Physiological and Morphological Changes on Chicken Meat *Campylobacter* Isolates

**DOI:** 10.3389/fmicb.2020.00503

**Published:** 2020-03-25

**Authors:** Gayani Kuriyawe Muhandiramlage, Andrea R. McWhorter, Kapil K. Chousalkar

**Affiliations:** School of Animal and Veterinary Sciences, University of Adelaide, Adelaide, SA, Australia

**Keywords:** *Campylobacter*, chlorine inactivation, poultry meat, cell damage, bacterial resuscitation

## Abstract

Broiler chickens frequently become colonized by *Campylobacter* species. As a consequence, *Campylobacter*, can enter the poultry meat supply chain and represents a significant risk for human public health. A number of on-farm biosecurity and processing measures are used to mitigate the load of *Campylobacter* on chicken meat. In many countries, chlorine is commonly used as a biocide in processing plants to reduce bacterial loads on poultry carcasses but there is limited evidence of its effectiveness on *Campylobacter*. In this study, 116 *Campylobacter* isolates (89 *C. jejuni* and 27 *C. coli*) were isolated from poultry meat carcasses prior to the inside/outside wash step and used in *in vitro* assays exploring the efficacy of chlorine. A high proportion of isolates exhibited MIC and MBC values of 128 ppm but organic material present in the broth likely affected this result. Thus, additional bactericidal assays (time kill and chlorine inactivation) were used to characterize the response of *C. jejuni* isolates to different concentrations of chlorine. At 10^6^ CFU, *C. jejuni* was found to be highly sensitive to concentrations of chlorine and was inhibited at low concentrations (0.2–2.0 ppm). At a higher bacterial load (10^8^ CFU), variation in the response of different *C. jejuni* isolates was observed. One isolate was growth inhibited at 1.8 ppm while another required 16 ppm. At 10^8^ CFU, *C. jejuni* could be resuscitated following exposure to chlorine highlighting a potential limitation of chlorine use. Analysis of UV leakage indicated that high chlorine concentrations resulted in increased 280 nm absorbance values suggesting bacterial membrane damage. Scanning electron and transmission electron microscopy were performed to characterize the morphological effects of chlorine exposure. Some effects of chlorine exposure included changes in shape (coccoid, or elongated), cellular degeneration, and shriveled bacterial cells. Interestingly, *C. jejuni cells* with normal morphology were also observed in the chlorine exposed group and represent a population of cells that could be resuscitated. This study is useful for the chicken meat industry and provides data for future optimization of chlorine use in reducing *Campylobacter* loads.

## Introduction

*Campylobacter* species are the most common cause of bacterial associated foodborne gastrointestinal disease in humans (Havelaar et al., 2015; Kirk et al., 2015). In 2010, of the 600 million global cases of foodborne related disease, 96 million were caused by *Campylobacte*r (Havelaar et al., 2015). The number of campylobacteriosis cases are increasing and current estimations of disease rates in Europe are 30–50 per 100,000 annually, 14–50 per 100,000 in North America, 112 per 100,000 in Australia, and 1512 per 100,000 in Japan (Connerton and Connerton, 2017). There are 26 known *Campylobacter* species and of these, *C. jejuni* and *C. coli*, are among the leading causes of campylobacteriosis (Kirk et al., 2015; Patrick et al., 2018). Humans can be exposed to *Campylobacter* through consumption of contaminated, untreated water and a wide variety of food items. Raw or under-cooked poultry meat and poultry meat products, however, are among the most frequently identified sources of *Campylobacter* (Hoffmann et al., 2017).

At hatch, broiler chicks are generally free from *Campylobacter* but acquire the bacteria in the growing sheds (Herman et al., 2003). Once a few birds become positive, the bacterium spreads horizontally within the flock (Jacobs-Reitsma et al., 1995; Herman et al., 2003) but significant variability in the total *Campylobacter* load has been observed between individuals in a given flock (Hansson et al., 2010). Transport stress from farm to the processing plant has also been linked with higher *Campylobacter* loads being shed in broiler feces (Stern et al., 1995; Whyte et al., 2001). As a consequence, chicken meat can become contaminated during various points of processing (Hermans et al., 2012). High loads of *Campylobacter* entering poultry meat processing plants have been linked with higher increased risk of carcass contamination (Lindblad et al., 2006; Hansson et al., 2007). A recent review revealed that depending on geographical location, between 19–100% of post-production poultry meat products can be contaminated with *Campylobacter* (Suzuki and Yamamoto, 2009). This level of contamination represents a significant public health risk through direct consumption of improperly cooked chicken meat or cross contamination of food preparation environments.

Globally, a number of different methods are used within the poultry meat industry to mitigate the pathogen load on the surface of whole chicken carcasses as well as meat pieces including “generally recognized as safe” (GRAS) chemicals, such as chlorine in the form of sodium hypochlorite, acidified sodium chloride, and peracetic acid (PAA). The use of each of these chemicals, however, varies widely from country to country. The use of PAA in the spin chill wash, for example, has been increasing in the United States (Walsh et al., 2018). The European Union has banned the use of most pathogen reduction chemicals except for water and lactic acid for health and safety reasons (Anonymous, 2004). In Australia and many Asian countries, however, chlorine continues to be the most commonly used poultry meat sanitizer (Anonymous, 2005; Chousalkar et al., 2019). Comparatively, chlorine has a lower cost than other sanitizers and along with ease of use may account for its continued application.

Chlorine is an oxidizing agent that has been shown to cause membrane permeabilization in both Gram negative (*Yersinia enterocolitica* and *Escherichia coli*) and Gram positive (*Listeria monocytogenes* and *Bacillus subtilis*) bacterial species (Virto et al., 2005). Multiple bacterial species, however, are known to have the ability to recover from chemical or environmental stress (Wesche et al., 2009), however, little is known about the resuscitation of *Campylobacter* after sub-lethal exposure to chlorine. The decontamination of poultry carcasses in the processing plant represents a significant challenge because of the constant presence of organic material being added to sanitizing solutions during processing. Increasing concentrations of organic material (such as residual fecal material, blood, skin, or feathers) reduces the total load of free chlorine in solution (LeChevallier et al., 1981; Virto et al., 2005).

Several studies have shown that chlorine wash steps in the poultry meat processing line leads to significant reductions in *Campylobacter* loads (Lu et al., 2019). To date, it has not been demonstrated whether the total *Campylobacter* load has an effect on the efficacy of the sanitizer. The objective of the current study was to isolate *Campylobacter* from poultry meat carcasses and characterize the physiological, morphological and cellular responses of these isolates to chlorine exposure using different concentrations of bacteria. An additional aim was to determine whether there was inherent variability in the chlorine sensitivity of chicken meat *Campylobacter* isolates and their resuscitation potential following exposure to chlorine.

## Materials and Methods

### Isolation of *Campylobacter*

The *Campylobacter* isolates used in this study were isolated during a separate study investigating the efficacy of sanitizers during chicken meat processing (Chousalkar et al., 2019). Briefly, chicken meat carcasses (15 birds from each of eight different broiler production sheds) were collected from two separate processing plants prior to the inside-outside wash step. Bacteria were isolated by massaging chicken carcasses (prior to sanitizer exposure) in buffered peptone water (BPW) (Oxoid, Australia). Two hundred microliters of BPW wash was spread plated onto modified charcoal-cefoperazone deoxycholate agar (mCCDA) (Oxoid, Australia) and incubated at 42°C in 10% CO_2_ for 48 h. Putative, *Campylobacter* isolates were sub-cultured once to obtain pure cultures, stored at −80°C in 5% glycerol, and further characterized using PCR. A total of 116 *Campylobacter* isolates were obtained.

In preparation for experiments, bacteria were resuscitated from freezing stocks on to Columbia sheep blood agar (SBA) (Oxoid, Thermo Scientific, Australia) and incubated at 42°C in 10% CO_2_ for 48 h. The *Campylobacter jejuni* ATCC 33291 strain was used as a control strain.

### Multiplex PCR Identification of *Campylobacter coli* and *Campylobacter jejuni*

*Campylobacter* isolates were further characterized using a multiplex PCR enabling the distinction of *Campylobacter coli* (*C. coli*) and *Campylobacter jejuni* (*C. jejuni*) strains. Bacterial DNA was extracted using 0.6% Chelex resin (BioRad, United States) according to the manufacturer’s instructions. Purified DNA was stored at −20°C until required.

The multiplex PCR method designed by Van et al. (2017) enabled the distinction of *C. coli* and *C. jejuni* strains. To detect *C. jejuni*, primers (Forward: 5′-ACT TCT TTA TTG CTT GCT GC-3′, Reverse: 5′-GCC ACA ACA AGT AAA GAA GC-3′) designed to the *hipO* gene were used. This reaction generated an amplicon of 323 base pairs. To distinguish *C. coli*, specific primers (Forward: 5′-GCT GCA CTT TTA AAT CCA G-3′, Reverse: 5′-CTT TGG TTT TAC AAT ATG AGC-3′) designed to the *glyA* gene were used. The *C. coli* reaction generated an amplicon of 186 base pairs.

The *C. coli* and *C. jejuni* multiplex PCR reaction was conducted using a total volume of 20 μL. Each reaction contained 100 ng DNA, 1× My Red Taq reaction buffer, 250 nM each *C. jejuni* forward and reverse primer, 500 nM each *C. coli* forward and reverse primer, 0.2 units of My Red Taq polymerase (Bioline, Australia), and nuclease free water. PCR cycling conditions were performed using a Biorad T100 thermocycler. The first step was an initial melt at 98°C for 1 min. The second step included 35 cycles of 98°C for 10 s, 59°C for 30 s, and 72°C for 30 s. A final extension was done at 72°C for 10 min. Products were electrophoresed using a 2% agarose gel.

### Determination of Minimum Inhibitory and Minimum Bactericidal Concentrations of Chlorine

The chlorine minimum inhibitory concentration (MIC) was determined for both *C. jejuni* (*n* = 89) and *C. coli* (*n* = 27) isolates. The MIC was determined using the broth microdilution method in nutrient broth number 2 (NB2) (Oxoid, Australia) according to the Clinical and Laboratory Standard Institute guidelines (Weinstein, 2018). Chlorine (4% Sodium hypochlorite, Sigma-Aldrich, Australia) concentrations ranging from 2 ppm to 1024 ppm (pH range 7.5–8.0) were prepared using 96 well round bottom microtiter plates (Thermo Scientific, Australia). *Campylobacter* inoculums were prepared using a 0.5 McFarland standard and were confirmed measuring the optical density at 600 nm to obtain 10^8^ CFU/mL. Subsequently, 10 μL of the *Campylobacter* inoculum (10^6^ CFU) was added into 990 μL of chlorine dilutions. The positive control was comprised of NB2 without chlorine but containing bacteria; the negative control was NB2 only. All isolates were tested in duplicate. *C. jejuni* ATCC 33291 was included as the experimental standard. MIC plates were incubated at 42°C in 10% CO_2_ for 20 h. The lowest concentration, which did not give visible bacterial growth was defined as the MIC. Isolates with disparate results in replicate wells were repeated.

After the MIC was determined, wells showing growth inhibition were drop plated on to SBA agar plates to determine the minimum bactericidal concentration (MBC). Briefly, 10 μL of broth from each well in MIC plate was drop plated on to SBA plates and incubated at 42°C in 10% CO_2_ for 48 h. The MBC was defined as the lowest bactericidal concentration of chlorine required to kill bacteria after incubation at 42°C in 10% CO_2_ for 20 h.

### Time Kill Assays

Time-kill assays were used to determine the susceptibility of *Campylobacter* isolates to chlorine. Two *C. jejuni* isolates, with disparate MIC values of 128 ppm (C1) and 16 ppm (C2), were selected for this experiment. To determine the effect of organic load, time-kill experiments were performed using both NB2 and 0.9% saline. Standard chlorine concentrations used in the poultry industry ranges between 8–10 ppm when contacting carcasses (Oyarzabal, 2005), hence, dilutions were prepared at half the standard (4 ppm), standard concentration (8 ppm), and twice the standard (16 ppm). The inoculum was prepared by suspending *Campylobacter* colonies in either NB2 or 0.9% saline and matching the turbidity of an 0.5 McFarland standard. Subsequently, 10 μL of *Campylobacter* inoculum was added to 990 μL of each chlorine dilution for a final bacterial concentration of 10^6^ CFU/mL and exposed to chlorine for 24 h at either 5°C and 25°C. Bacterial counts were determined at specific intervals post exposure (2, 20, 60, 90, 120, 240, 480, and 1440 min). Serial 10-fold dilutions in 0.9% saline were immediately prepared and 10 μL was drop plated onto SBA and incubated at 42°C in 10% CO_2_. Time kill assays were conducted in triplicate and repeated two times.

### Chlorine Inactivation Assay

Microbial resistance to chlorine was evaluated using the chlorine inactivation assay. For these experiments, six *C. jejuni* isolates were randomly selected based on the MIC and MBC results. Low (0.2–2 ppm) and high (2 ppm–256 ppm) chlorine concentrations were prepared in 0.9% saline. To determine whether bacterial load had an effect on the efficacy of chlorine, two inoculum doses, 10^8^ CFU/mL and 10^6^ CFU/mL, were used. Bacteria were exposed to chlorine for 2 min at 25°C. Bacterial counts were obtained by drop plating 10 μL of serial 10-fold dilutions on to SBA and incubating at 42°C in 10% CO_2_ for 48 h. Normal saline without chlorine was used as the bacterial growth control. All isolates were tested in duplicate and each experiment was repeated twice.

### Bacterial Resuscitation

After 2 min of exposure to chlorine, 100 μL of all treatment and control groups in the inactivation assay were inoculated into 900 μL of Preston broth (nutrient broth number 2 with *Campylobacter* selective supplement) (Oxoid, Australia) and incubated at 42°C in 10% CO_2_ for up to 48 h. After 24 h and 48 h incubation, 10 μL of each treatment was drop plated on to SBA to determine if the *Campylobacter* isolate had recovered from exposure to chlorine. Each resuscitation experiment was performed with two replicates and was repeated twice.

### Leakage of UV-Absorbing Material

Quantifying the amount of UV absorbent material is often used as a measure for bacterial membrane damage (Virto et al., 2005; McKenzie et al., 2016). To characterise chlorine induced membrane damage, 300 μL of each treated and untreated samples were collected after the 2-min exposure (inactivation assay) and centrifuged at 6000 × *g* for 10 min. UV absorbencies were read at 260 nm and 280 nm with a spectrophotometer (ClarioStar, BMG Lab Tech, Australia). All six isolates were tested in duplicates at 25°C and were repeated.

### Preparation of Bacteria for Microscopy

Electron microscopy was performed to characterise the morphological and cellular changes of *C. jejuni* isolates after exposure to 8 ppm chlorine for 2 min. After chlorine exposure, the bacterial pellets of both control and treatment was obtained by centrifuging at 8000 × *g* for 10 min. The pellet was resuspended in normal saline and washed twice to remove excess chlorine. Subsequently, the pelleted cells were fixed in electron microscopy fixative (4% glutaraldehyde in 0.1 M sodium cacodylate buffer (pH 7.2). Sample preparation and microscopy were conducted at Adelaide Microscopy, The University of Adelaide, Adelaide SA, Australia.

### Transmission Electronic Microscopy (TEM)

The bacterial pellet was washed twice in PBS with 4% sucrose for 10 min, followed by post-fixation with 1% Osmium tetroxide for 1 h. After washing, bacteria were dehydrated in a graded ethanol series (70, 95, and 100%), substituted with propylene oxide, embedded in LX112 epoxy resin, and placed in a 60°C oven for 24 h to polymerize the resin. Ultrathin (60–80 nm) sections were prepared by cutting resin-fixed pellets with an RMC Power Tome Ultramicrotome and double stained with uranyl acetate and lead citrate. Sections were examined utilizing a TEI Tecnai G2 Spirit Bio TWIN at 20–120 kV and imagining was done via in – column Olympus-SIS Veleta CDD camera.

### Scanning Electron Microscopy (SEM)

*C. jejuni* cultures were filtered through 0.2 μm membrane filters (Whatman, United States), washed with PBS + 4% sucrose and fixed with 2% osmium tetroxide for 45 min. After washing, cells were dehydrated in a graded ethanol series (70, 90, and 100%). The filters were then dried with 1:1 ratio of 100% ethanol: hexamethyldilazane for 30 min followed by 100% hexamethyldilazane for 10 min. Cultures were air dried, mounted on carbon stubs, and sputter coated with platinum. Specimens were examined under FEI DualBeam (FIB/SEM) scan electron microscope, operated at 10 kv.

### Statistical Analysis

Experimental data for bacterial counts and UV absorbance data are presented as mean ± standard error. One-way Analysis of Variance (ANOVA) and Two-way ANOVA followed by Tukey’s multiple comparison test were used to determine statistical differences of the effects of chlorine on *Campylobacter* bacterial load. Prevalence data were analyzed using Fisher’s exact test.

All statistical analyses were performed using either SPSS Version 25 (IBM, United States) or GraphPad Prism Version 8.3.0 (GraphPad Software, Inc., United States). In all cases, a *P*-value of < 0.05 was considered statistically significant.

## Results

### PCR Typing of *Campylobacter* spp. Isolated From Chicken Meat

Putative *Campylobacter* colonies were collected from positive chicken carcasses and tested using the *C. jejuni* and *C. coli* multiplex PCR. A total of 116 isolates were obtained and of these, 76.7% (89/116) were positive for the *C. jejuni* amplicon while 23.2% (27/116) were positive for the *C*. *coli* amplicon. The percentage of *C. jejuni* isolates collected from the chicken meat samples was significantly higher (*P* < 0.05) than *C. coli*.

### *Campylobacter* Susceptibility to Chlorine: MIC and MBC Determination

**FIGURE 1:**
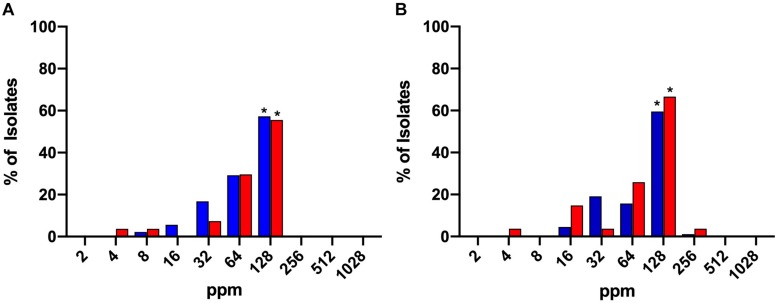
MIC **(A)** and MBC **(B)** values of chlorine (2–1028 ppm) for *C. jejuni* (blue) and *C. coli* (red) strains isolated from chicken meat. A significant majority of both isolates exhibited MIC or MBC values of 128. *Denotes statistical significance (*P* < 0.05).

The bactericidal activity of chlorine was tested over a range of concentrations from 2 to 1028 ppm. The MICs and MBCs obtained for both *C. jejuni* and *C. coli* isolates are shown in Figures 1A,B, respectively. The majority of both *C. jejuni* (57.3%) and *C. coli* (55.5%) isolates exhibited an MIC of 128 ppm and this result was significant (*P* < 0.05). MBC values were similar for 59.5% of *C. jejuni* and 66.6% of *C. coli* isolates exhibiting an MBC of 128 ppm. No significant difference was observed for MIC or MBC values between *Campylobacter* species.

### Chlorine Time Kill Kinetics

Organic material present in the NB2 media used for MIC and MBC tests likely reduced the biocidal effect of chlorine on *Campylobacter.* To confirm this, time kill curves were performed using both NB2 and 0.9% saline. Two *C. jejuni* isolates with disparate chlorine MIC values were selected for this experiment. Isolate C1 had an MIC of 128 ppm while the MIC for C2 was 16 ppm.

**FIGURE 2:**
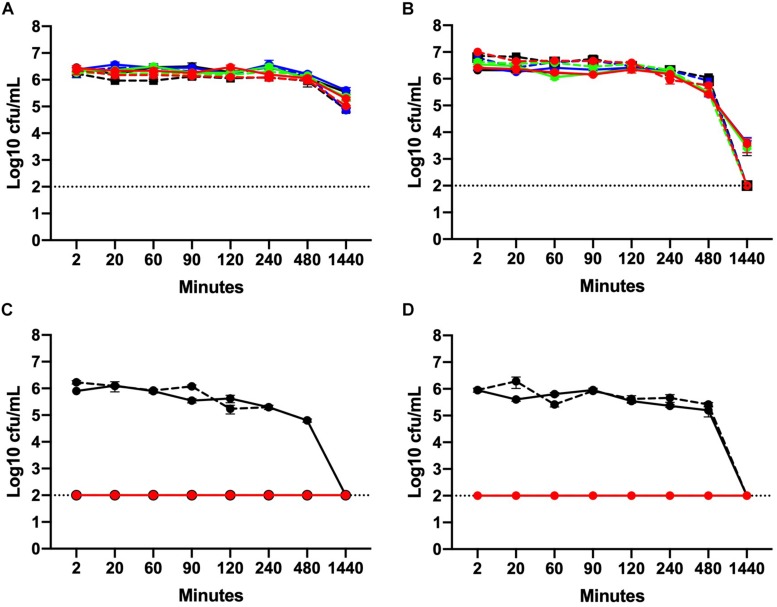
Chlorine time kill curves of two *C. jejuni* isolates with MIC values of 128 ppm (C1) and 16 ppm (C2). Isolates were suspended in either NB2 **(A, B)** or 0.9% saline **(C, D)** at 5°C **(A, C)** or 25°C **(B, D)**. *C. jejuni* isolates were exposed to three different concentrations of chlorine 4 ppm (blue), 8 ppm (green), and 16 ppm (red). Bacteria suspended in either NB2 broth or 0.9% saline were included as control (black). Isolate C1 is designated using solid lines and C2 hashed lines. No significant effect of chlorine was observed for bacteria suspended in NB2 **(A, B)** but a significant effect was observed at all chlorine concentrations for both *C. jejuni* isolates suspended in 0.9% saline (*P* ≤ 0.001) **(C, D)**. The dotted line indicates limit detection for culturable bacteria.

Data are presented as mean log_10_ CFU/mL *C. jejuni*. At 5°C, no significant effect of chlorine was observed on the total *C. jejuni* load over time in NB2 (Figure 2A). At 25°C in NB2, the total bacterial load did not vary significantly between 2 min and 8 h of exposure at any of the chlorine concentrations tested (Figure 2B). A significant reduction (*P* ≤ 0.001) in bacterial loads was observed at 25°C for all treatment groups after 24 h of exposure. A 3-log reduction was observed at 24 h for the C1 treatment groups while a 4-log reduction was observed all C2 treatment groups. Interestingly, the reduction in bacteria observed at 25°C after 24 h of did not occur at 5°C.

In 0.9% saline, the culturability of both *C. jejuni* isolates C1 and C2 was completely inhibited following exposure to chlorine. After 2 min of exposure, no culturable bacteria (either C1 or C2) were obtained from all three chlorine concentrations at either 5 or 25°C (Figures 2C,D). No significant difference in the mean bacterial load was observed for the control groups between 2 min and 8 h. After 24 h, however, culturable bacteria were not detected at either temperature.

### Inactivation Kinetics of Chlorine on *Campylobacter jejuni*

**FIGURE 3:**
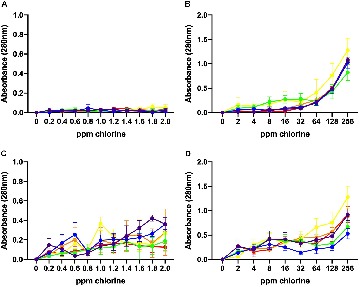
Inactivation kinetics of chlorine on six different *C. jejuni* isolates: C1 (red), C2 (orange), C3 (yellow), C4 (green), C5 (blue), and C6 (purple). 10^6^ CFU **(A, B)** and 10^8^ CFU **(C, D)** of each isolate were exposed to low **(A, C)** and high **(B, D)** concentrations of chlorine. At 10^6^ CFU, all *C. jejuni* isolates were highly sensitive to chlorine at all concentrations **(A, B)**. The higher inoculum, 10^8^ CFU, a significant effect of chlorine concentration was observed for both the low **(C)** (*P* < 0.001) and high **(D)** (*P* < 0.001) range. Individual variation between isolates was observed in response to chlorine. Isolate, C3, was the most sensitive to chlorine and was inhibited at the lowest concentration (1.8 ppm). This result was significant (*P* < 0.01).

Based on results from the time kill experiments, two minute inactivation experiments were conducted for the six *C. jejuni* isolates using low (0.2 – 2.0 ppm) and high (2 – 256 ppm) concentrations of chlorine (Figure 3). At 10^6^ CFU/mL, all six isolates were highly sensitive to chlorine at both low and high concentrations and were not culturable following a 2-min exposure (Figures 3A,B). Interestingly, at this load, none of the *C. jejuni* isolates were cultured from the lowest chlorine concentration of 0.2 ppm (Figure 3A).

At 10^8^ CFU/mL, a significant effect of chlorine was observed for both the low (*P* < 0.001) and high (*P* < 0.001) range of concentrations tested. C3, and C4 were the most sensitive *C. jejuni* isolates. At 1.8 and 2.0 ppm of chlorine, culturable C3 bacteria were no longer detected while C4 exhibited a 3 log_10_ reduction in the total number of culturable bacteria. C3, and C4 exhibited significantly lower (*P* < 0.001) bacterial loads at chlorine concentrations between 1.4–2.0 ppm compared with C2, C5, and C6 isolates. At the low range of chlorine concentration, no significant change in the number of culturable C6 was observed.

A significant effect of chlorine was observed over the high concentration range (*P* < 0.001). Consistent with the low range data, isolate C3 was not culturable at 2 ppm or higher. Significant variability was observed amongst the isolates in their response to chlorine (*P* < 0.001). Isolate C6 retained the highest bacterial loads up to 8 ppm where it was no longer culturable. C1 and C5 remained culturable at 8 ppm but there was no significant difference between the bacterial loads. No isolate was cultured from chlorine suspensions ranging from 16–256 ppm.

### Recovery of *Campylobacter* After Chlorine Treatment

After 2 min of exposure to different chlorine concentrations, 100 μL of each *Campylobacter* isolate were added to resuscitation media to determine sub-lethal injury. The number of non-culturable isolates and whether they were resuscitated is shown in Table 1. Non-culturable *Campylobacter* from the 10^6^ CFU/mL chlorine treatment groups were not resuscitated after either 24 or 48-h incubation in Preston broth.

**TABLE 1:** Resuscitation of *C. jejuni* after inactivation at different chlorine levels.

	Bacterial concentration (10^6^ CFU/ml)			Bacterial concentration (10^8^ CFU/ml)		
		
Chlorine (ppm)	Unculturable isolates	Resuscitated isolates 24 h	Resuscitated isolates 48 h	Unculturable isolates	Resuscitated isolates 24 h	Resuscitated isolates 48 h
2	6/6	0/6	0/6	1/6	1/1	1/1
4	6/6	0/6	0/6	2/6	2/2	2/2
8	6/6	0/6	0/6	6/6	6/6	6/6
16	6/6	0/6	0/6	6/6	2/6	2/6
32	6/6	0/6	0/6	6/6	1/6	1/6
64	6/6	0/6	0/6	6/6	1/6	1/6
128	6/6	0/6	0/6	6/6	0/6	0/6
256	6/6	0/6	0/6	6/6	0/6	0/6

Bacterial resuscitation in Preston broth was also performed for 10^8^ CFU/mL *C. jejuni* treatment groups following exposure to chlorine. At 2 ppm, 1/6 isolate was non-culturable but was resuscitated after both 24 and 48-h incubation in broth. Similarly, at 4 ppm, 2/6 isolates were non-culturable following chlorine exposure and were both resuscitated. All *C. jejuni* isolates were resuscitated in broth after exposure to 8 ppm chlorine. After exposure to both 32 and 64 ppm chlorine, 6/6 isolates were non-culturable but only one isolate was resuscitated at both concentrations. It should be noted that this was the same isolate. None of the *C. jejuni* isolates were culturable after exposure to 128 or 256 ppm and could not be resuscitated.

### Leakage of UV-Absorbing Substances Caused by Chlorine Treatment

**FIGURE 4:**
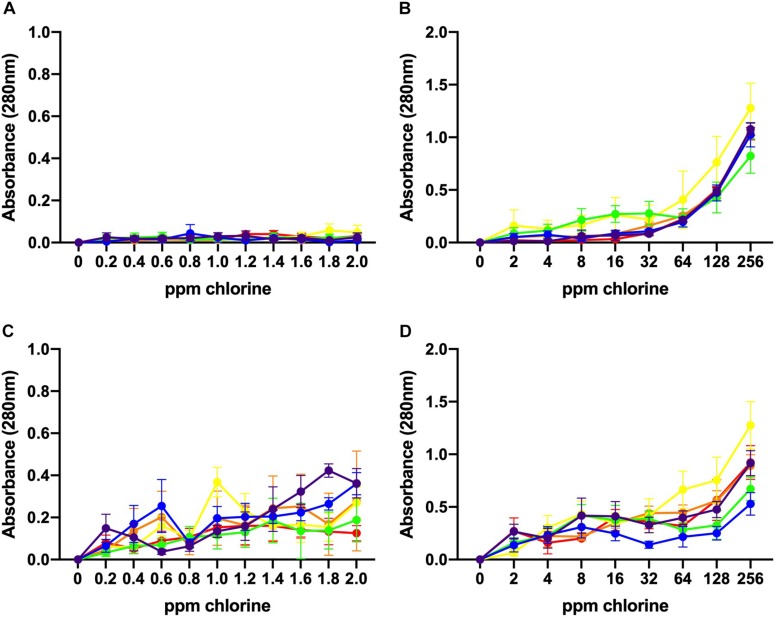
Leakage of UV-absorbing substances caused by chlorine treatment. Absorbance (280 nm) values for six *C. jejuni* isolates: C1 (red), C2 (orange), C3 (yellow), C4 (green), C5 (blue), and C6 (purple) at low, 10^6^ CFU/mL **(A, B)**, and high, 10^8^ CFU/mL **(C, D)**, loads for over low **(A, C)** and high **(B, D)** chlorine concentrations. At 10^6^ CFU/mL, membrane damage following exposure to low concentrations of chlorine was minimal as UV absorbance values observed were <0.150 **(A)**. Increasing concentrations of chlorine resulted in significantly higher (*P* < 0.001) 280 nm absorbance values for the 10^6^ CFU/mL *C. jejuni* suspension **(B)**. At low chlorine concentrations, a significant increase in absorbance at 280 nm was also observed for the10^8^ CFU/mL suspension (*P* < 0.001) **(C)**. Similar results were observed for the high chlorine range **(D)**. A significant effect of concentration (*P* < 0.01) was detected for the 10^8^ CFU/mL suspensions with increasing UV absorbencies observed between 2 – 256 ppm (Figure 4D).

The values of UV absorbance at 280 nm of the supernatants collected from 10^6^ to 10^8^ CFU/mL *C. jejuni* suspensions after exposure to low and high chlorine concentrations are shown in Figure 4. At 10^6^ CFU/mL, membrane damage following exposure to low concentrations of chlorine (0.2–2 ppm) was minimal as UV absorbance values observed were less than 0.150 (Figure 4A) and no significant effect of chlorine was observed. Increasing concentration of chlorine resulted in significantly higher (*P* < 0.001) 280 nm absorbance values for the 10^6^ CFU/mL *C. jejuni* suspension (Figure 4B).

Higher absorbencies were observed for the 10^8^ CFU/mL *C. jejuni* suspension over the low concentration range as compared with the 10^6^ CFU/mL suspension (Figure 4C). At low chlorine concentrations, a significant increase in absorbance at 280 nm was observed for the10^8^ CFU/mL suspension (*P* < 0.001). Similar results were observed for the high chlorine range. A significant effect of concentration (*P* < 0.01) was detected for the 10^8^ CFU/mL suspensions with increasing UV absorbencies observed between 2 – 256 ppm (Figure 4D). Variation in UV absorbance values were observed between strains especially when the bacterial load was high but no significant difference between isolates was detected. Of note, isolate C3, which was inactivated at the lowest concentrations exhibited the highest absorbance values at both low (Figure 4B) and high (Figure 4D) bacterial concentrations. The results obtained at 260 nm (data not shown) were similar to those obtained at 280 nm.

### Morphological Changes Caused by Chlorine Treatment

**FIGURE 5:**
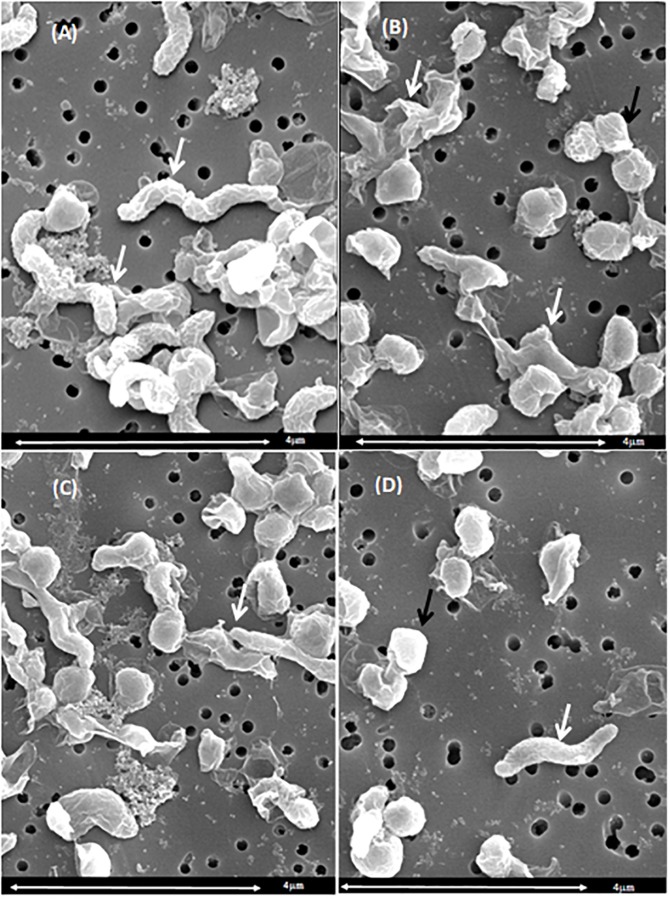
Morphological changes of *C. jejuni* following chlorine exposure: SEM. A *C. jejuni* isolate was exposed to either saline only **(A)** or 8 ppm chlorine **(B–D)** for 2 min. Bacteria with normal spiral morphology can be observed in the saline treatment group **(A**, white arrows). Exposure to chlorine resulted in damaged cell membranes **(B**, white arrow), coccoid morphology **(B–D)**, and stretched morphology **(C**, white arrow). The typical *C. jejuni* spiral morphology was also observed **(D**, white arrow).

**FIGURE 6:**
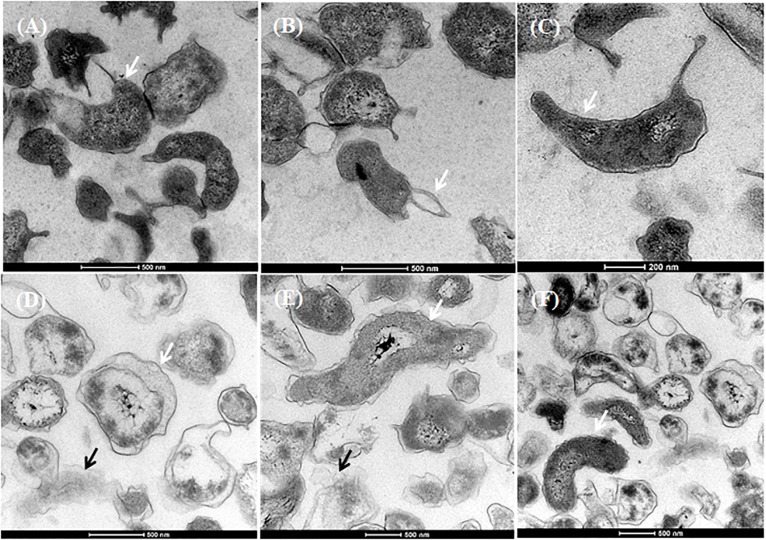
Morphological changes of *C. jejuni* following chlorine exposure. TEM images of *C. jejuni* exposed to either saline only **(A–C)** or 8 ppm chlorine **(D–F)**. Normal morphology was observed in the 0.9% saline treatment group **(A–C**, white arrows). Chlorine exposed bacteria exhibited detached cytoplasm **(D**, black arrow; **E**, white arrow), leakage of cytoplasm **(D**, white arrow; **E**, black arrow). *C. jejuni* cells with normal cytoplasmic morphology were also observed **(F**, white arrow) in the chlorine treated group.

Based on the UV absorbance data, we investigated the morphological changes in *C. jejuni* after exposure to chlorine using SEM (Figure 5) and TEM (Figure 6). The cell structure of *C. jejuni* following a 2-min exposure to 8 ppm of chlorine was substantially altered. Under SEM, *C. jejuni* cells appeared either coccoid or exhibited a stretched and shriveled morphology (Figures 5B–D). Loss of integrity and complete destruction of the cell membrane was also observed (Figure 5B). Unaffected cells exhibiting normal *C. jejuni* morphology were also observed in the chlorine treatment group (Figure 5D, white arrow). TEM analysis of *C. jejuni* exposed to chlorine revealed that treated cells were not dense as cells in the control (Figures 6D–F). The bacterial cells in various stages of degeneration were also observed. The cytoplasm of some bacterial cells was detached from the cell membrane (Figure 6D, white arrow). Cells with severely damaged the cell membranes and extruded cytoplasm were also frequently observed (Figures 6D,E).

## Discussion

In the present study, *Campylobacter* strains were isolated from poultry meat carcasses prior to the inside/outside wash step at the processing plant. Of the 116 *Campylobacter* isolates obtained, 76.7% were identified as *Campylobacter jejuni*. While only a single isolate was collected from each positive carcass, our results are consistent with previous study demonstrating that *C. jejuni* is more common than *C. coli* on chicken meat carcasses (Anonymous, 2010). Another recent Australian report, however, indicated the *C. coli* was more common (Walker et al., 2019). The strains isolated from the present study were then used in experiments designed to characterize their sensitivity to chlorine.

In some countries, chlorine is often used in chicken meat processing plants with the objective to reduce levels of *Campylobacter* and *Salmonella* (Anonymous, 2005; Chousalkar et al., 2019). The MIC experiments revealed that 97.5% of *C. jejuni* and 92.6% *C. coli* isolates required chlorine levels higher than 8 ppm to inhibit their growth completely. Furthermore, MBC results demonstrated that 57.3% of *C. jejuni* and 55.5% of *C. coli* required 128 ppm of chlorine to inhibit the growth completely. The interpretation of these results is limited because due to the presence of chlorine demanding material in the NB2 broth. Furthermore, the free available chlorine is constantly monitored to ensure the consistent bactericidal effects. Therefore, to further explore the response of the *C. jejuni* isolates to chlorine other bactericidal tests were used in the presence and absence of chlorine demanding material.

The time kill experiments conducted here revealed that the effect of chlorine over time was significantly impacted by the presence of organic material in the broth. No significant difference in bacterial loads was observed at any of the chlorine concentrations prepared in NB2. Irrespective of the treatment effect, the bacterial counts of both isolates declined after 24 h incubation at 25°C. This may be due to the fastidious growth requirement of *Campylobacter* and the restriction of growth below 30°C (Park, 2002). In contrast, both C1, C2 isolates exhibited one log_10_ reduction at 5°C after 24 h. This suggested that regardless of the chlorine effect, *Campylobacter* remained viable when incubated at 5°C. The rapid transformation of *C. jejuni* in to the viable but non-culturable (VBNC) form at 25°C and its viability at low temperatures has been described earlier (Jang et al., 2007). Further work is necessary to assess the chlorine resistance and survival of *Campylobacter* on chicken carcass under these parameters. Viability of *Campylobacter* at 5°C in presence of chlorine is an important observation for the industry as the chill tanks in processing plants are most often maintained at this temperature. Interestingly, when 0.9% saline was used instead of NB2, both C1, C2 *Campylobacter* isolates were highly sensitive to chlorine at all concentrations. These results could be attributed to the negative effect of chlorine neutralizing substance in the reaction media (NB2). Previous studies have shown that Gram-negative bacteria are resistant to chlorine in the presence of organic matter (Virto et al., 2005). Organic matter reduces the free available chlorine in a solution ultimately providing protection to bacteria (LeChevallier et al., 1981).

Inactivation assays were performed to determine the chemical efficacy bacterial concentrations over a broad range of chlorine concentrations. The low range of concentrations (0.2–2.0 pmm) were used to determine the chlorine sensitivity of the *Campylobacter* isolates used in this study. The high range of concentrations (2.0 ppm to 256 ppm) incorporated the levels of chlorine used globally in poultry processing plants. Chlorine was more effective in reducing *Campylobacter* levels when the bacterial inoculum was low (10^6^ CFU/ml). Interestingly, when the bacterial load was 10^8^ CFU/ml, reduction in *Campylobacter* level was lower. Our findings suggest that the efficacy of chlorine is highly dependent on a load of *C. jejuni* in the reaction media. This may be due to the reduction of free available chlorine required to inactivate all bacteria. The mechanism behind this difference, however, requires further study. In the present study, strain variation in chlorine sensitivity was also observed among *C. jejuni* isolates. The strain variation in response to chlorine treatment has been previously shown (Blaser et al., 1986). Usually, broiler birds harbor high levels of *Campylobacter* in the fecal material as well as in the crop and cross contaminate carcass during the processing (Newell and Fearnley, 2003). Loads of *Campylobacter* entering processing plants can vary significantly and has been previously reported to range between 5 log_10_ CFU/mL to 8 log_10_ CFU/mL of carcass rinse (Bashor et al., 2004).

The resuscitation of *C. jejuni* after exposure to chlorine suggested that it is possible to revive bacteria after a sub-lethal injury caused by chlorine exposure. Notably, resuscitation was observed only, when the *Campylobacter* concentration was 10^8^ CFU/mL in the reaction media. About 83.3% of *C. jejuni* isolates were able to resuscitate after exposure to chlorine at 8 ppm. Additionally, 66.6% of isolates were resuscitated at 4 ppm. Isolates were not resuscitated when the bacterial load was low (10^6^ CFU/ml) even in the lowest concentration of 0.2 ppm. This may be due to irreversible cell injury caused by chlorine when the bacterial load was high. There was no difference in the resuscitation of bacteria with extended incubation in broth for up to 48 h. This suggested that reversible cellular injury could be repaired within 24 h of enrichment. The bacterial recovery in response to chlorine could also vary from strain to strain. This strain variation of recovery could be due to the genetic variation among the *Campylobacter* strains in order to overcome the chemical stress (Peyrat et al., 2008). Our findings suggested that the bacteria could overcome the sub-lethal injury if provided with sufficient recovery time under appropriate enrichment conditions (Wesche et al., 2009). The recovery of *Campylobacter* in liquid media after exposure to various stressors such as nutrient and heat stress has been previously demonstrated (Bovill and Mackey, 1997) but there is a lack of reports demonstrating *Campylobacter* resuscitation after chlorine exposure.

To demonstrate the extent of bacterial membrane damage due to chlorine treatment, the leakage of the intracellular substances was measured by UV absorbance. When the outer membrane of a Gram-negative bacteria is damaged, the intracellular substances such as lipopolysaccharides, lipids, phospholipids, and periplasmic enzymes leads to leaking out through the membrane and that may disrupt membrane permeability (Wesche et al., 2009). In the present study, the UV absorbance values increased as chlorine concentrations increased. This was, however, also influenced by the number of bacteria (10^6^ vs. 10^8^ CFU/mL). Our findings can be compared with a previous study where authors reported increasing UV absorbance values from *Escherichia coli* following exposure to increasing concentrations of chlorine (Virto et al., 2005).

The severity of cellular damage after chlorine exposure were further investigated using both TEM and SEM. SEM observations revealed that bacterial cell shape varied markedly after exposure to chlorine. Chlorine stressed bacteria exhibited a loss of their distinct spiral shape. Similar degenerative changes have been previously reported for heat stressed *C. jejuni* (Tangwatcharin et al., 2006). The cell fragmentation and loss of polysaccharide capsule in the present study are evidence of irreversible lethal injury. The SEM confirmed the loss of the typical spiral *C. jejuni* morphology following chlorine stress, with the coccoid form being more dominant in the treated cells. Similar morphological changes have been described for *Campylobacter* cells exposed to antibiotics (Xie et al., 2011). It is important to note that following chlorine exposure, some *C. jejuni* cells exhibited normal morphology under SEM. Therefore, based on the SEM findings and resuscitation assay, it can be concluded that after exposure to chlorine, the non-injured or sub-lethally injured bacterial cells could be repaired if the appropriate enrichment conditions are provided.

## Conclusion

In conclusion, the results of the present study revealed that, although chlorine is effective in reducing *C. jejuni* contamination, it is unable to eliminate it completely. This study was performed *in vitro*; hence it is essential to perform similar work using chicken meat carcasses. The *Campylobacter* isolates used in this study exhibited varying sensitivities to the chlorine concentrations tested. This could be a result to genetic variation among individual adaptive stress tolerance response mechanisms. Further studies are required to investigate the molecular mechanisms behind the strain variation. If present in high numbers, *Campylobacter* could be resuscitated after chlorine exposure that highlights the limitation of chlorine use. Ultimately, a combined chemical decontamination strategy beneficial for the chicken meat industry but further study is required. The data obtained in this study is useful for the chicken meat industry for further optimizing the use of chlorine in reducing *Campylobacter* loads.

## Data Availability Statement

The data sets generated during this study are available by request from the corresponding author.

## Author Contributions

GM, AM, and KC designed all the experiments. KC and AM supervised all the experiments. GM performed all the data analyses and *Campylobacter* PCR, MICs, MBCs, SEM and TEM, and prepared the first draft of the manuscript. GM and AM conducted the chlorine inactivation, UV-absorbance assays, and resuscitation experiments. AM and KC provided the assistance with the manuscript preparation and performed the edits.

## Conflict of Interest

The authors declare that the research was conducted in the absence of any commercial or financial relationships that could be construed as a potential conflict of interest.
